# Effectiveness of a Central Discharge Element Sock for Plantar Temperature Reduction and Improving Comfort

**DOI:** 10.3390/ijerph18116011

**Published:** 2021-06-03

**Authors:** Alfonso Martínez-Nova, Víctor Manuel Jiménez-Cano, Juan Miguel Caracuel-López, Beatriz Gómez-Martín, Elena Escamilla-Martínez, Raquel Sánchez-Rodríguez

**Affiliations:** 1 Nursing Department, Podiatric Clinic of the University of Extremadura CPUEX, 10600 Plasencia, Spain; victormajc@unex.es (V.M.J.-C.); jcaracue@alumnos.unex.es (J.M.C.-L.); bgm@unex.es (B.G.-M.); escaelen@unex.es (E.E.-M.); rsanrod@unex.es (R.S.-R.); 2 Nursing Department, University of Extremadura, Plasencia (Centro Universitario de Plasencia), Avda, Virgen del Puerto 2, 10600 Plasencia, Spain

**Keywords:** foot posture, skin care, foot, health, socks, comfort

## Abstract

U-shaped plantar cushions could help reduce stress affecting the central forefoot without the need for an orthosis, but they are yet to be integrated as an element in socks. The objective of this study was to verify the effectiveness of a sock with a central discharge element in terms of plantar temperature and comfort. The sample comprised 38 subjects (13 men and 25 women). Their plantar temperatures were measured with a thermographic camera in a basal situation and after each of two 10-minute walks around an indoor circuit during which they wore either control or experimental socks at random (the same design, weight, and fiber, but with the plantar cushioning element added). After the walks, each subject responded to a comfort questionnaire (five-point Likert scale), blindly scoring the two socks. The highest temperatures (28.3 ± 2.7 °C) were recorded in the zone of the second and third metatarsal heads. With the experimental socks, the observed temperature increase in the central forefoot zone was significantly less than with the control socks (31.6 vs. 30.6 °C, *p* = 0.001). The subjects found the experimental socks to be more comfortable than the controls (4.63 ± 0.5 vs. 4.03 ± 0.5, *p* < 0.001). The discharge element included in the experimental socks was effective since it reduced the contact zones and excess friction with the ground, thereby lessening overheating by more than 1 °C. Furthermore, the experimental socks were perceived as being more comfortable by the subjects who had mild and occasional foot discomfort.

## 1. Introduction

Hiking is an outdoor activity which has increased greatly in society due to its numerous beneficial physical and mental effects [1]. Nonetheless, it can also be the cause of certain musculoskeletal lesions of low or moderate importance [2]. One example is metatarsalgia, i.e., pain in the plantar zone of the metatarsus, which is one of the most common reasons for foot pathology consultations [3,4] and can be a limiting factor for this type of walking. Although the central forefoot is of preponderant importance in the normal plantar pressure map [5], metatarsalgia is usually associated with increased pressure peaks on the second and third metatarsals [6,7], which can lead to the appearance of associated dermal or keratotic lesions. In podiatry, a common treatment for the relief of over pressure in this pathology is the application of temporary cushioning elements (doughnut-shaped, U-shaped, or C-shaped felt pads) that are arranged to cover the entire metatarsal zone except for the sector overloaded and in pain, which remains on a somewhat higher plane and therefore with less participation in gait [8,9,10]. However, these cushioning elements must be replaced very frequently, every 3–5 days, as their efficacy is short-term [11,12]. They can also be integrated into an orthopedic insole [13], although they will still be elements external to the user’s usual clothing, as well as being subjected to intense wear and therefore needing to be renewed with a certain periodicity.

It would hence be desirable to have cushioning elements for the metatarsal zone that could be part of the usual apparel, such as the socks, since their properties (knitted structure, fabric, and yarn) could play a role in skin damage and blister formation [14] and have already been shown to be effective in reducing plantar over pressure [15,16]. In the current state of this technology, there are various types of socks fabricated with different elements or with different configurations that provide potential beneficial health effects. The focus of the existing models is, however, not specifically on the plantar part of the sock, so there is no simple and economical solution to protect the metatarsals and mitigate the pain and discomfort usually caused by their pathologies. Therefore, the authors have proposed socks with a cushioning element integrated into the body of the garment to provide it with these beneficial effects [17]. This element consists of a padded zone (made with different threads woven onto the base of the sock) with a U-shaped anterior discontinuity or opening in the zone of the second and third metatarsal heads (MTHs). The intention with this model is to reduce the support and pressure that this zone is subjected to, leading to a more dorsiflexion posture of the central metatarsals.

Thermography is used in medicine in several research fields, such as allergology, dermatology, physical therapy, and dentistry, as well as in a wide range of sports, to assess the physiological responses of the skin under different conditions, which can lead to the discovery of vascular diseases or aid the in the screening of breast cancer [18]. The analysis of the temperature of the foot’s plantar surface is an indirect way to evaluate plantar load, which has shown a good capacity to predict the vertical, triaxial, and shear components of those loads [19,20]. In this way, analysis with infrared thermography could capture the temperature differences between the regions of interest in the foot, and identify the zones with greater biomechanical participation while walking (as hotter zones), and therefore those that support a greater load. With this technology, it has been possible to determine that, both before and after a race, the highest temperature in the forefoot zone corresponds to the central region [21].

Keeping the zone of the second and third MTH in a slightly higher plane than the rest of the foot by means of the cushioning element integrated into the sock would lead to a reduction in support and friction during the middle and propulsive support phases of gait, and this could be identified by a lower temperature while walking. This reduction of load could have the effect of increasing comfort and alleviating the load on the zone, and reducing the pain or discomfort associated with sustained over pressure. Nonetheless, the proposed sock had not yet been tested to evaluate its effectiveness, so our working hypothesis was that the addition of a discharge element in the plantar surface of the sock would lessen the increase in temperature at the central forefoot due to the reduction in plantar pressure values. Therefore, the objective of the present study was to make an initial evaluation of plantar temperatures and compare them with the temperatures after two 10-minute walks, one while wearing normal commercial socks (control) and another wearing socks with the same characteristics but with an integrated metatarsal cushioning element (experimental). A further intention was to evaluate the comfort perceived by the subjects during the walks.

## 2. Materials and Methods

### 2.1. Participants

The convenience sample consisted of 38 subjects (13 men and 25 women) with a mean age of 28.1 ± 10.6 years, mean weight of 68.5 ± 12.2 kg, mean height of 170 ± 8.3 cm, and mean body mass index of 24.1 ± 3.2 kg/m^2^. The sample was recruited from the students and academic staff of the University Centre of Plasencia. The study was conducted between 8–20 April 2021. The study fulfilled the requirements of the Helsinki declaration, and was approved by the Bioethics and Biosafety Commission of the University of Extremadura (ID: 180//2020) and registered on clinicaltrials.gov (NCT04697914).

The inclusion criteria for participation in the study were: subjects (a) between 18 and 60 years old, (b) with a structurally normal foot, with a foot posture index from −4 to +9, and (c) not suffering from significant or disabling pain (allowed characteristics were slight and occasional pain in the central forefoot, and the presence of overload or superficial hyperkeratosis in the zone of the second and/or third MTH). The exclusion criteria were: subjects who presented with (1) intense and frequent plantar pain, (2) obvious gait or balance disturbances, or (3) the inability to perform the two 10-minute walks normally.

### 2.2. Instruments and Procedure

#### 2.2.1. Thermographic Analysis

Before the images were taken, the subjects were acclimated to the conditions of the room. The temperature and relative humidity were measured with a FLIR MR77 meter (Flir systems), with the means being 20 ± 0.5 °C and 50 ± 5%, respectively. Temperature analyses were carried out with a Flir E60bx thermographic camera (Flir systems; resolution: 320 × 240 pixels, temperature range: −20 °C to 1200 °C, thermal sensitivity: less than 0.045 °C between pixels). The subjects were requested not to drink alcohol or hot drinks at least 4 hours before the trial. Sport or intense physical activity were not permitted the day before the assessment [22]. For the plantar thermographic measurements, the camera was placed 1 m away from the feet, in accordance with the protocol described by Gatt et al. [23]. The subject took off their own socks, lay down on a stretcher, and placed their feet in slight dorsiflexion 5–10 cm apart. A black screen was placed above the feet to avoid the reflection of heat from the rest of the body. One minute was allowed to pass before taking the photograph to avoid the image being conditioned by previous activity or the manipulation of the foot. After taking this prior plantar image, the subject put on the pair of socks (control or experimental, as selected at random).

#### 2.2.2. Socks

**Figure 1:**
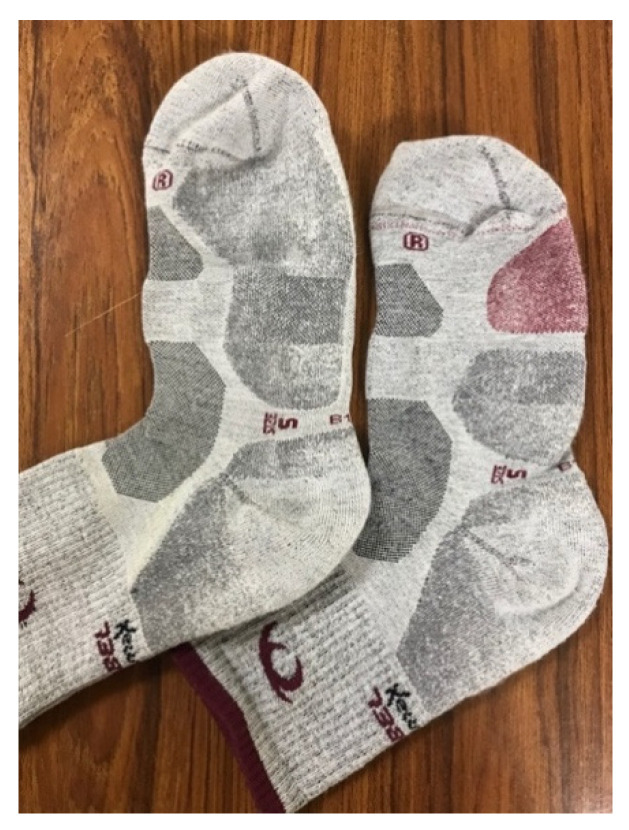
Detail of the two socks, control (**left**) and experimental (**right**).

**Figure 2:**
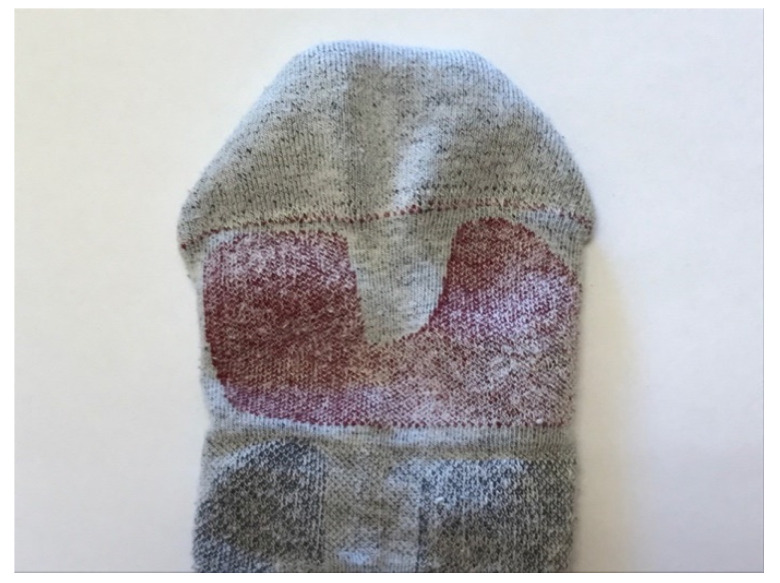
Metatarsal cushioning of the experimental sock.

The control sock was the Lurbel Tierra^®^ trekking model, made with 50% Regenactiv (cellulose-based fiber with ionic chitosan particles), 25% Cool-Tech, 17% Ionized Polyamide, and 8% Lycra, with a weight of 60 g for size M (Figure 1). The experimental sock was fabricated following the same model (fabric, knitted, fibers, design, weight, and fit; Figure 1 and Figure 2) as the control, but with the addition of the cushioning element (3 mm) based on Utility Model ES1247681U [17].

#### 2.2.3. Walking Protocol

After putting the socks on, the subject proceeded to walk for 10 minutes around an indoor circuit, which was the same for all participants. The subject then returned to the measurement room, lay down on the stretcher, the black screen was again put in place, and another one minute was allowed to pass before taking the second photograph. The subject then put on the other pair of socks, and walked for another 10 minutes around the same indoor circuit, after which they went back to the measurement room to have a third photograph taken following the identical protocol as before.

#### 2.2.4. Comfort Questionnaire

After the two walks, the subjects responded to a comfort questionnaire in which they scored the comfort of the two socks on a scale of 1 to 5 (1: very uncomfortable, 2: uncomfortable, 3: neutral, 4: comfortable, and 5: very comfortable). At no time did they know which were the experimental and which were the control socks, since they scored the socks according to their red (experimental, Figure 1 right) or grey (control, Figure 1 left) details.

### 2.3. Analysis of Thermographic Variables

**Figure 3:**
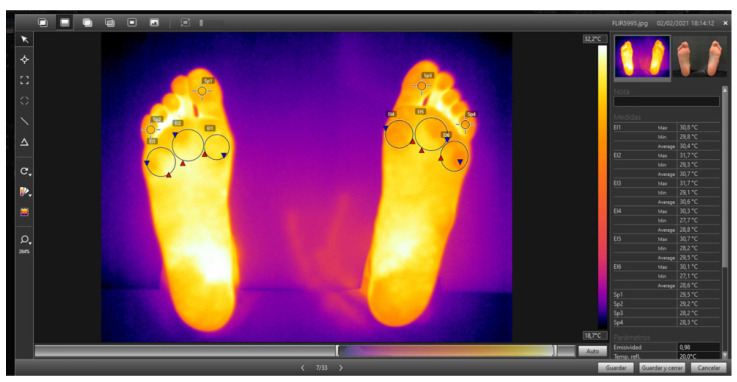
Thermographic image. Detail of the black background (top, right), with the anatomical zones analyzed. The software yields the upper, lower, and mean temperatures for each zone.

To measure the temperature with the associated software (Flir Tools), the forefoot (the area in which the experimental cushioning element was located) was divided into 5 zones: (1) the first metatarsal head, (2) the second and third MTH, (3) the fourth and fifth MTH, (4) the first toe, and (5) the fifth toe (Figure 3). The mean temperature for the entirety of each zone analyzed was calculated as an average of all pixels in a marked area, as this was a more robust measure [24]. To avoid bias, the researcher responsible for the thermographic analysis was blinded in the study.

### 2.4. Statistical Analysis

To maintain data independence [25], the data of the left foot (chosen at random) were entered for statistical analysis. After verifying the fit of the data to normality (Kolmogorov–Smirnov test, *p* > 0.05 in all cases), a descriptive analysis and Student’s t-test were applied to determine the prior overall temperature and differences by sex. Since the sample fulfilled the assumption of sphericity (*p* > 0.05 in all of the 3-layer comparisons), a repeated measures ANOVA (3 × 3, post-hoc Bonferroni) was applied to the prior measurement and the measurements after walking with the control and the experimental socks. All statistical calculations were performed using the SPSS version 22.0 (IBM, Armonk, NY, USA) program package (UEX campus license), setting a significance level of 5% (*p* < 0.05).

## 3. Results

Initially, for the overall sample, the highest temperature (28.3 ± 2.7 °C) was located in the zone of the second and third MTH, and the lowest (25.9 ± 3.4 °C) in that of the ball of the fifth toe. There were no significant differences in the initial temperatures between men and women (*p* > 0.05 in all cases, Table 1).

The repeated measures ANOVA showed that, in all five zones analyzed, the temperature increased significantly between the initial and the two subsequent measurements with each sock model (*p* < 0.001 in all cases, Table 2). Under the second and third MTH, the temperature was lower (by 1 °C) for the experimental socks than for the controls (*p* < 0.001).

With respect to comfort, the subjects scored the control socks at 4.03 ± 0.5 and the experimental socks at 4.63 ± 0.5, with the difference being statistically significant (*p* < 0.001).

## 4. Discussion

The central zone of the forefoot (second and third MTH) presented the highest temperatures in the base conditions and with the control socks. This is coherent with previous observations by Escamilla-Martínez et al. [21], in which the temperature in this zone was measured at 28.7 ± 2.2 °C in conditions prior to a race, a value remarkably similar to that obtained in the present study (28.3 ± 2.7 °C). Although foot temperature can be influenced by various biomechanical variables, the second and third MTH would be warmer due to their greater participation in the statics and dynamics of gait [26]. The data are also consistent with the highest pressure values in normal feet, which, in the forefoot, are located in said central zone [27], reflecting the great relevance of this zone in gait biomechanics.

Our subjects experienced a rise in temperature in all of the zones analyzed after both walks compared to their prior temperature. This temperature increase after the physical activity (even though only very lightly aerobic) was to be expected since an increase in body temperature due to movement leads to an increase in skin temperature, including that of the feet [28]. In this experiment, the time and the circuit to be walked were controlled, but not the gait cadence, since, as Reddy et al. [29] conclude, there are no differences in final temperature when walking at different gait cadences. After the walk with the experimental socks, due to the lower temperature observed in the central zone, the hottest zone was that of the first MTH head. This could be indicative of the sock’s effectiveness, since, if one extrapolates the lower temperature to the reduction in plantar pressure [19], the cushioning of the central zone of the forefoot has managed to transfer load to the medial zone (the first MTH).

To date, technological development in socks has mainly focused on incorporating new fibers with potential beneficial effects, such as fluorine [30] or chitosan [31], that improve localized thermal conditions. The addition of other fibers based on noble metals such as copper [32] or nanosilver [33], or on bioceramics, which have been shown to have antimicrobial [34] and thermoregulatory effects [21], could help reduce the risk of skin lesions such as fissures and small wounds [35]. In contrast, the design of the plantar part of the sock has not received much technological attention, and it is clear that a redesign of its structure could have beneficial effects in reducing pressure and increasing comfort.

The sock has been identified as an external conditioning factor in the development of lesions [36], and zones of the foot which are sensitive or have some pre-existing pathology may predispose an individual to the development of dermal lesions [37]. Therefore, the use of the proposed experimental sock in subjects with metatarsalgia, plantar hyperkeratosis, blisters, or plantar hypersensitivity would protect the central zone from excessive friction with the shoe and could help prevent pain or the appearance of dermal lesions. Furthermore, this sock model was perceived by the sample as being more comfortable than the control sock, which corroborates the positive effect of the proposed model.

Socks with metatarsal cushioning could be very useful for trekking activities and for long, multi-stage walks in which foot lesions are frequent. Chicharro et al. [2] found that up to 74% of walkers doing the Camino de Santiago (Saint James Way) pilgrimage presented with blistering lesions after completing some of the stages, which could lead to their abandoning of the activity due to the intense pain. Furthermore, one of the most frequent zones (18.3%) where these blisters appeared was that of the second and third MTH. In this sense, a U-shaped cushion is the usual choice to apply for the central MTH [12], and it seems to be more effective in reducing plantar pressure than the C-shape [38]. In addition, this U-shape, being open at the front of the sock, prevents the formation of window oedema [39], which can be a complication of doughnut-shaped cushioning [40].

This study has important clinical implications, since individuals who practice hiking or trekking could institute planned prevention prior to the activity. We consider this sock morphology able to relieve high plantar pressures with a more long-lasting element. Thus, a correct choice of technical socks with a cushioning element and with a three-dimensional plantar shape that is adapted to an individual’s anthropometric characteristics, foot posture, or to any pre-existing light discomfort (such as mild metatarsalgia, plantar keratoses, or index-minus metatarsal formulas) could help reduce the load on the central zone of the forefoot, increase comfort, and reduce the risk of the appearance of lesions that might hinder or interrupt the activity.

## 5. Limitations of the Study

The present study is subject to some limitations. The main one is that the walks the subjects performed were of short duration, around an indoor circuit, and of a very light intensity. Thus, the results must be understood in this context. An experiment is being designed to verify these data with an outdoor walk of at least 1 hour to extrapolate the data to the reality of an activity like walking.

## 6. Conclusions

The U-shaped metatarsal discharge element of the experimental sock led to a lesser temperature increase under the second and third MTH. This may be due to reduction in load and less contact of this zone with the shoes. The subjects also perceived the experimental socks as being more comfortable. Individuals with pain or sensitivity in the central forefoot zone could therefore use them as a preventive measure to avoid dermal lesions.

## Figures and Tables

**Table 1:** Initial temperatures in all zones, and differences by sex.

	Mean ± SD (°C)	Gender	Mean ± SD (°C)	*p* Value
First MTH	28.0 ± 2.8	Men Women	27.0 ± 3.2	0.115
			28.5 ± 2.5	
Second/third MTH	28.3 ± 2.7	Men Women	27.3 ± 3.0	0.141
			28.7 ± 2.5	
Fourth/fifth MTH	27.6 ± 2.7	Men Women	26.9 ± 2.9	0.250
			28.0 ± 2.6	
Hallux	26.1 ± 3.7	Men Women	25.3 ± 4.1	0.319
			26.6 ± 3.4	
Lesser toes	25.9 ± 3.4	Men Women	25.5 ± 3.8	0.555
			26.2 ± 3.2	

*t* test for independent samples.

**Table 2:** Repeated measures ANOVA.

	Base	Control	Experimental	W Mauchly (Sig)	Pillai’s Trace	*p* Value
	Mean (°C)					
First MTH	28.1	31.1	31.2	0.986 (*p* = 0.788)	0.701	<0.001 *
Second/third MTH	28.3	31.6	30.6	0.977 (*p* = 0.622)	0.656	<0.001 *†
Fourth/fifth MTH	27.6	30.5	30.6	0.974 (*p* = 0.623)	0.600	<0.001 *
Hallux	26.2	29.5	29.7	0.971 (*p* = 0.588)	0.579	<0.001 *
Lesser toes	25.9	29.2	29.3	0.999 (*p* = 0.977)	0.566	<0.001 *

* Difference between the base and the post-walking measurements. † Difference between the base and the post-walking measurements and between the two socks.
